# Interfering with hyaluronic acid metabolism suppresses glioma cell proliferation by regulating autophagy

**DOI:** 10.1038/s41419-021-03747-z

**Published:** 2021-05-13

**Authors:** Tao Yan, Xin Chen, Hua Zhan, Penglei Yao, Ning Wang, He Yang, Cheng Zhang, Kaikai Wang, Hong Hu, Jiafeng Li, Jingxian Sun, Yu Dong, Enzhou Lu, Zhixing Zheng, Ruotian Zhang, Xiaoxiong Wang, Jichao Ma, Ming Gao, Junyi Ye, Xinzhuang Wang, Lei Teng, Huailei Liu, Shiguang Zhao

**Affiliations:** 1 Department of Neurosurgery, First Affiliated Hospital of Harbin Medical University, Harbin, 150001, Heilongjiang Province China; 2 Key Colleges and Universities Laboratory of Neurosurgery in Heilongjiang Province, Harbin, 150001, Heilongjiang Province China; 3 Institute of Neuroscience, Sino-Russian Medical Research Center, Harbin Medical University, Harbin, 150001 Heilongjiang Province China; 4 North Broward Preparatory School, 7600 Lyons Rd Coconut Creek, Orlando, FL 33073 USA; 5 Department of Neurosurgery, Second Affiliated Hospital of Zhejiang University, Hangzhou, 310009 Zhejiang Province China; 6 Department of Neurosurgery, Shenzhen Samii Medical Center, Shenzhen, 518118 Guangdong Province China; 7 Biomolecular Science Center, Burnett College of Biomedical Sciences, University of Central Florida, Orlando, FL 32816 USA; 8 Department of Neurosurgery, Shenzhen University General Hospital, Shenzhen, 518100 Guangdong Province China

**Keywords:** Cancer metabolism, Drug development, Cancer genomics

## Abstract

The tumor microenvironment plays an important role in tumor progression. Hyaluronic acid (HA), an important component of the extracellular matrix in the tumor microenvironment, abnormally accumulates in a variety of tumors. However, the role of abnormal HA accumulation in glioma remains unclear. The present study indicated that HA, hyaluronic acid synthase 3 (HAS3), and a receptor of HA named CD44 were expressed at high levels in human glioma tissues and negatively correlated with the prognosis of patients with glioma. Silencing HAS3 expression or blocking CD44 inhibited glioma cell proliferation in vitro and in vivo. The underlying mechanism was attributed to the inhibition of autophagy flux and maintaining glioma cell cycle arrest in G1 phase. More importantly, 4-methylumbelliferone (4-MU), a small competitive inhibitor of Uridine diphosphate (UDP) with the ability to penetrate the blood-brain barrier (BBB), also inhibited glioma cell proliferation in vitro and in vivo. Thus, approaches that interfere with HA metabolism by altering the expression of HAS3 and CD44 and the administration of 4-MU potentially represent effective strategies for glioma treatment.

## Introduction

Glioma is the most common intracranial malignant tumor. Although patients undergo adjuvant chemotherapy and radiotherapy after surgical resection, the survival time of patients is still short, and the disability rate is high^1^. A better understanding of the mechanism and development of tumorigenesis is very important for effectively controlling glioma progression. The tumor microenvironment is a key factor contributing to cancer cell survival and it is a regulatory network composed of many cells and factors that maintain the growth of tumor cells^2^. Strategies targeting the tumor microenvironment and its mediators effectively inhibit tumor cell growth^3^.

Hyaluronic acid (HA) is an important extracellular matrix component in the tumor microenvironment^4^. HA is a linear polysaccharide consisting of D-glucuronic acid and N-acetyl-D-glucosamine^5^. HA is derived from tumor cell secretions or the interaction between tumor cells and stromal cells and is present at high levels in the microenvironment of many types of tumors^6^. The HA family includes high molecular weight (HMW) HA (>1,000,000 Da), low molecular weight (LMW) HA, and oligosaccharides (o-HA) (∼400–20,000 Da). LMW-HA refers to HA with a molecular weight between HMW-HA and o-HA. HMW-HA can be degraded into LMW-HA or o-Ha when the body is subjected to an injury or a pathological insult, such as cancer^7^. HAs of different molecular weights exert different biological functions. For instance, HMW-HA possesses antiangiogenic and immunosuppressive activities, whereas LMW-HA induces inflammation and is related to the angiogenesis, survival, growth, and metastasis of tumors^8^. The accumulation of HA is closely related to the prognosis of patients with malignant tumors, such as malignant breast, prostate, ovarian, and lung tumors^9^. Other factors that are involved in HA metabolism (precursors of HA synthesis, HA synthases, HA receptors, and hyaluronidase) are associated with tumor development^10^. According to previous studies, HA synthases, including HAS1, HAS2, and HAS3, play crucial roles in the process of HA synthesis. CD44 is an important HA receptor that promotes the metastasis, proliferation, and angiogenesis of invasive tumors and accelerates HA degradation^11,12^. Inhibition of CD44 activity induces the apoptosis of invasive tumors^13^. UDP is a precursor required for HA synthesis, and 4-methylumbelliferone (4-MU), which passes through the blood-brain barrier (BBB), is a competitive inhibitor of UDP^14,15^. Based on this evidence, HA and its receptors play important roles in tumor development. However, less is known regarding the role and molecular mechanisms of abnormal HA accumulation in glioma, and further studies are needed. Here, we examined the effect and mechanism of HA metabolism on glioma by interfering with HA metabolism through the suppression of production of the HA precursor, the expression of HA synthase, and the binding of HA with its receptor.

Autophagy is essential for maintaining tumor growth^16^. HA has a clear relationship with reactive oxygen species (ROS)^17^, and ROS is closely related to autophagy. Importantly, ROS are crucial regulators of autophagy under various conditions^18^. HA has been shown to regulate cell survival and growth through the PI3K/AKT/mTOR pathway, which is a typical signaling pathway inducing cell autophagy^19^. In addition, in patients with osteoarthritis (OA), HA regulates autophagy by altering the miRNA expression profile^20^. These evidences prompted us to explore whether abnormal HA metabolism affects glioma cell proliferation by regulating autophagy. Alterations in HA metabolism induced by silencing HAS3, blocking its binding with the receptor CD44, or administering 4-MU inhibited autophagy flux, arrested the cell cycle at G1 phase, and subsequently inhibited glioma cell proliferation in the present study. In conclusion, interfering with the synthesis of HA or blocking binding to its receptor may be a potential therapy for glioma treatment.

## Results

### HA, HAS3, and CD44 are increased in glioma tissues and negatively correlated with the prognosis of glioma

**Fig. 1:**
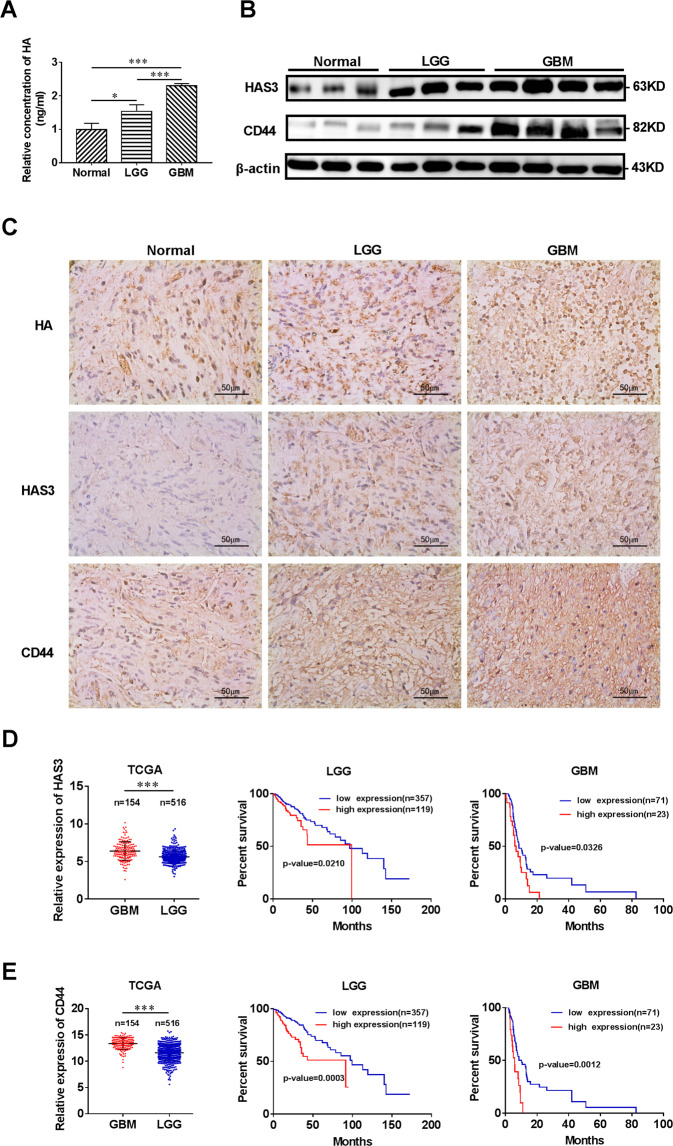
Hyaluronic acid, HAS3, and CD44 are increased in glioma tissues and negatively correlated with the prognosis of glioma. **A** Relative concentration of HA in human glioma tissues of different grades and normal brain tissues by ELISA. **B** Representative images of IHC staining for HA, HAS3, and CD44 in human glioma tissues of different grades and normal brain tissues. Scale bar: 50 μm. **C** Relative levels of the HAS3 and CD44 proteins in human glioma tissues of different grades and normal brain tissues. **D**–**E** Relative expression of HAS3 and CD44 mRNA in patients with LGG and GBM from TCGA. Survival curves of HAS3 and CD44 for patients with LGG and GBM from TCGA database. The data are presented as the mean ± SD; *P < 0.05, **P < 0.01, and ***P < 0.001, ****P < 0.0001.

HA levels were first detected in glioma tissues to investigate whether abnormal HA metabolism is associated with glioma. The HA level was significantly increased in tissues from patients with different grades of gliomas compared with normal tissues (Fig. 1A, C). Based on the abnormal accumulation of HA in patients with different grades of gliomas, we questioned whether factors related to HA metabolism are associated with glioma growth. HA-related synthases (HAS1, HAS2, and HAS3) and the receptor CD44 play important roles in the synthesis and biological function of HA, therefore, bioinformatics analysis was performed to analyze the expression of HA synthases and CD44, the results indicated that HAS1 expression was negatively correlated with the DFS time of patients with high-grade glioma (Supplementary Fig. 1A); however, the correlation was not significant for patients with LGG. In addition, HAS1 was expressed at higher levels in patients with LGG than in patients with GBM. Interestingly, although HAS3 and HAS2 expression were upregulated in patients with GBM compared with patients with LGG, only HAS3 expression negatively correlated with the DFS time in both LGG and GBM patients (Supplementary Fig. 1B; Fig. 1D). Moreover, in view of HAS1, HAS2, and HAS3 expression in glioma, we over-expressed HAS1 and knocked down HAS2 in glioma cell lines, the results demonstrated the over-expression of HAS1 or the silencing of HAS2 did not significantly affect the viability of glioma cells (Supplementary Fig. 1C). Therefore, we focused on HAS3 rather than HAS1 and HAS2 in subsequent experiments. CD44 is an important receptor for HA. CD44 expression was also upregulated and negatively correlated with the prognosis of patients with LGG and those with GBM (Fig. 1E). Therefore, HAS3 and CD44 were chosen for further analysis of HA metabolism in glioma. In addition, our research confirmed the significantly increased expression levels of HAS3 and CD44 in LGG and GBM tissues compared with normal tissues (Fig. 1B, C). Overall, HA, HAS3, and CD44 levels are significantly increased in glioma tissues and may play important roles in glioma progression.

### Interfering with HAS3 and CD44 suppresses glioma proliferation in vitro and in vivo

**Fig. 2:**
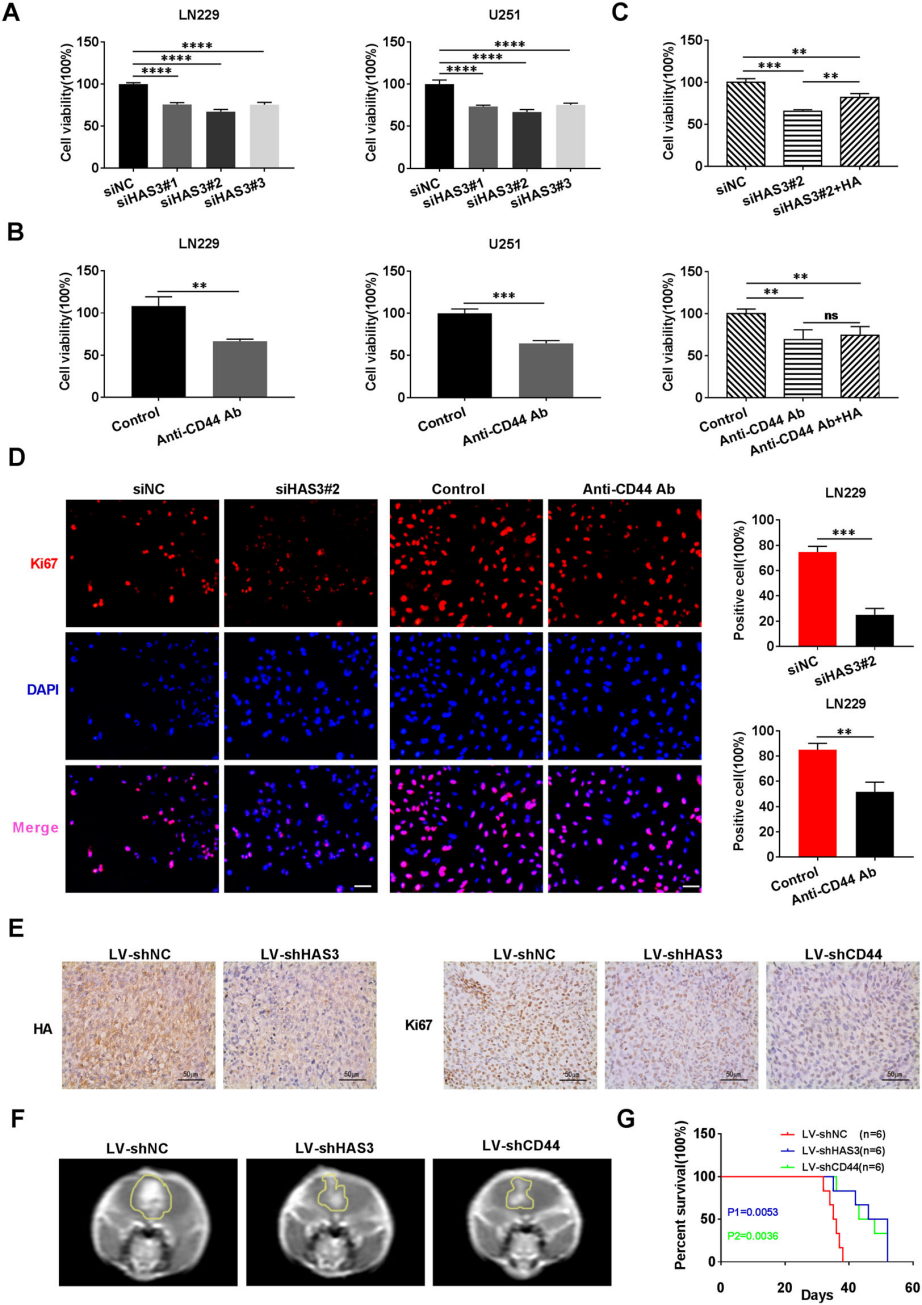
Treatments interfering with HAS3 and CD44 suppress glioma cell proliferation in vitro and in vivo. **A**–**B** Viability of U251 and LN229 glioma cells transfected with the HAS3 siRNA or treated with a CD44 antibody for 48 h. **C** Viability of U251 glioma cells transfected with the HAS3 siRNA or cultured with a CD44 antibody (6 µg/ml), followed by treatment with HA (25 µg/ml) for 48 h. **D** Levels of the Ki67 protein in LN229 glioma cells were detected using immunofluorescence staining after transfection with the HAS3 siRNA or treatment with a CD44 antibody for 48 h. Scale bar: 50 μm. **E** Representative images of IHC staining for HA in orthotopic xenograft tumors from the control and lentivirus HAS3 siRNA stably transfected glioma cell groups. Representative images of IHC staining for Ki67 in orthotopic xenograft tumors from the control and lentivirus HAS3 siRNA or lentivirus CD44 siRNA stable transfection glioma cell groups. Scale bar: 50 μm. **F**–**G** Representative MRI of orthotopic xenograft tumors and survival curves of the control and lentivirus HAS3 shRNA or lentivirus CD44 shRNA stably transfected glioma cell groups. P1: P-value for the comparison of shNC and shHAS3, P2: *P*-value for the comparison of shNC with shCD44. LV-shNC: negative control lentivirus, LV-shHAS3: HAS3-knockdown lentivirus. LV- shCD44: CD44-knockdown lentivirus. The data are presented as the mean ± SD; *P < 0.05, **P < 0.01, and ***P < 0.001, ****P < 0.0001.

We next explored the effects of interfering with HA metabolism mediated by inhibiting HAS3 or CD44 on glioma progression. First, the relative expression of HA, HAS3, and CD44 were determined in five glioma cell lines (LN229, U251, U87, T98, A172) and HUVEC cells. The results confirmed the relative expression levels of HA, HAS3, and CD44 in glioma were higher than those in HUVEC cells (Supplementary Fig. 1D–G). HA synthesis was inhibited by HAS3 silencing in U251 and LN229 glioma cell lines (Supplementary Fig. 2A). Notably, siRNAs were used to silence HAS3; alternatively, CD44 antibody was added to the culture media of glioma cells. The results of the MTT assay showed that the inhibition of HAS3 or treatment with CD44 antibody significantly decreased the viability of U251 and LN229 cells (Fig. 2A, B). Exogenous HA reversed this effect of HAS3 knockdown. In addition, the addition of exogenous HA also partially reversed the decline in cell viability caused by low concentrations (3 µg/ml) of the CD44 antibody, but did not reverse the effect of high concentrations of the CD44 antibody (6 µg/ml) in glioma cells (Fig. 2C, Supplementary Fig. 2B). A potential explanation for this result is that the CD44 antibody at a high concentration fully inhibits the binding of exogenous HA to CD44. Moreover, inhibition of HAS3 or treatment with the CD44 antibody decreased the expression of Ki67 in U251 or LN229 glioma cells, respectively (Fig. 2D and Supplementary Fig. 2C). To further verify the pro-survival effect of HAS3 and CD44, we over-expressed HAS3 and CD44 in glioma cell lines. However, the overexpression of HAS3 and CD44 did not significantly increase the activity of U251 and LN229 cells (Supplementary Fig. 2D). One possible explanation is that HAS3 and CD44 are already highly expressed in gliomas, and therefore, increasing their expression has no obvious effect on glioma proliferation. Next, we established stable U251 cell lines with knockdown of HAS3 and CD44 to further analyze the effects of HAS3 and CD44 on glioma cell viability and proliferation in vivo. As shown in Supplementary Fig. 2E, HAS3 and CD44 expression were significantly decreased in glioma cells transfected with the HAS3 siRNA and CD44 siRNA, respectively, compared with control glioma cells. A subcutaneous tumor model and an orthotopic tumor model were then established in nude mice using the stable U251 cell lines. The inhibition of HAS3 or CD44 in vivo significantly decreased the glioma volumes, extended the survival time of mice, and downregulated Ki67 expression compared with controls (Fig. 2E–G, Supplementary Fig. 2F–G). As expected, silencing of HAS3 also reduced the production of HA in vivo (Fig. 2E). Overall, the inhibition of HAS3 and CD44 decreased glioma cell proliferation in vitro and in vivo.

### Interfering with HAS3 and CD44 blocks autophagy flux in glioma

**Fig. 3:**
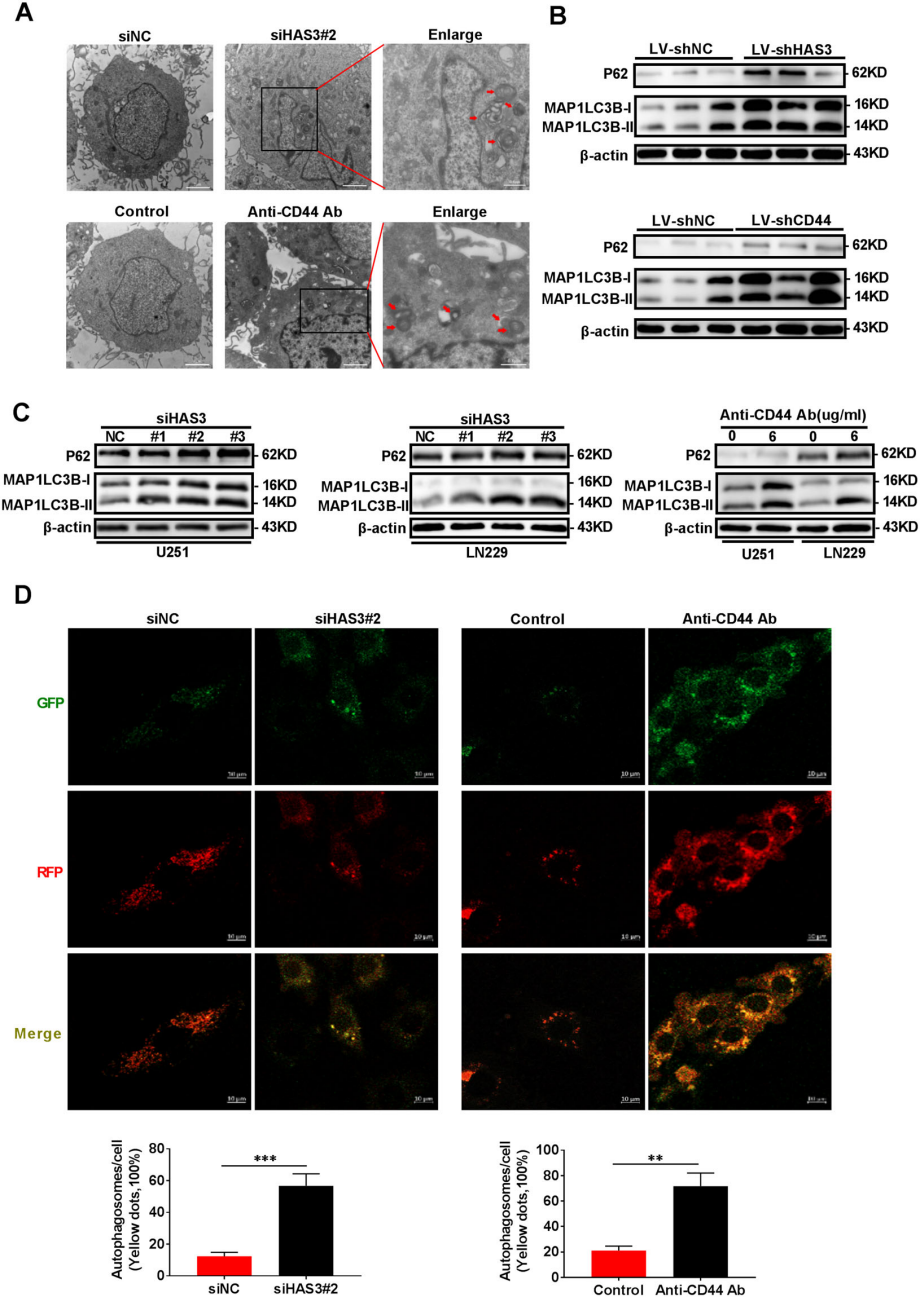
Treatments interfering with HAS3 and CD44 block autophagy flux in vitro and in vivo. **A** TEM images of U251 glioma cells transfected with the HAS3 siRNA or treated with a CD44 antibody for 48 h. The scale bars represent 2 μm in the original image, and the scale bars represent 0.8 μm in the enlarged image. **B** Relative levels of the P62 and MAP1LC3B proteins in the orthotopic xenograft tumors from the control and lentivirus HAS3 shRNA or lentivirus CD44 shRNA stably transfected glioma cell groups. **C** Relative levels of the P62 and MAP1LC3B proteins in U251 and LN229 glioma cells transfected with the HAS3 siRNA or treated with a CD44 antibody for 48 h. **D** Results of the GFP-RFP-LC3 fluorescence assay using U251 glioma cells transfected with the HAS3 siRNA or treated with a CD44 antibody for 48 h. Scale bar: 10 μm. The data are presented as the mean ± SD; *P < 0.05, **P < 0.01, and ***P < 0.001, ****P < 0.0001.

According to a previous study, HA is correlated with ROS, and ROS is closely associated with cell autophagy, and HA modulates the activity of the PI3K/AKT/mTOR pathway, which is also associated with autophagy. In addition, HA regulates cell autophagy in patients with OA. Based on these findings, we hypothesized that strategies interfering with HA metabolism would affect glioma growth by regulating autophagy. We transfected the HAS3 siRNA or added CD44 antibody to U251 glioma cells to test this hypothesis. Then, the number of autophagic vesicles was quantified using TEM. HAS3 silencing or treatment with the CD44 antibody increased the number of autophagic vesicles in glioma cells (Fig. 3A). Western blot results showed increased levels of MAP1LC3B-II and P62 in vitro and in vivo (Fig. 3B, C). The simultaneous increase in MAP1LC3B-II and P62 levels have been reported to indicate the blockade of autophagy flux in cells^21^. GFP-RFP-LC3 fluorescence assays were used to evaluate autophagy flux and confirm the effects of altered HA metabolism on autophagy flux. As expected, HSA3 silencing or the CD44 antibody treatment increased the number and intensity of yellow fluorescent dots in glioma cells compared with cells in the control group (Fig. 3D). This evidence further suggests that autophagy flux is blocked after HAS3 silencing or the binding of HA to CD44 receptors is inhibited in glioma cells. Moreover, exogenous HA reversed the effect of HAS3 silencing on autophagy (Supplementary Fig. 3A). Based on these results, treatments interfering with HA metabolism by silencing HAS3 or the application of an antibody against CD44 blocks autophagy flux.

### The blockade of HAS3 and CD44 combined with autophagy inhibitors exerts a synergistic inhibitory effect on glioma

**Fig. 4:**
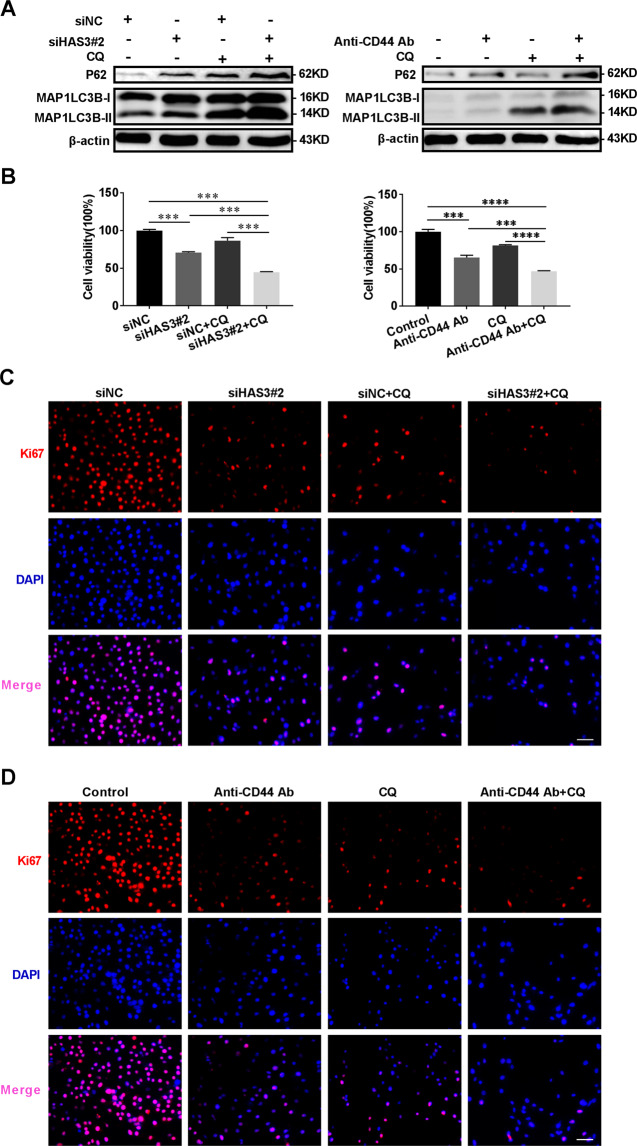
Treatments interfering with HAS3 and CD44 combined with autophagy inhibitors exert synergistic effects on glioma cell viability and autophagy levels. **A** Relative levels of the P62 and MAP1LC3B proteins in U251 glioma cells transfected with the HAS3 siRNA or cultured with a CD44 antibody, followed by treatment with CQ (30 μmol/L) for 48 h. **B** Viability of U251 glioma cells transfected with the HAS3 siRNA or cultured with a CD44 antibody, followed by treatment with CQ (30 μmol/L) for 48 h. **C**–**D** Levels of the Ki67 protein in U251 glioma cells were detected using immunofluorescence staining after transfection with the HAS3 siRNA or culture with a CD44 antibody, followed by treatment with CQ (30 μmol/L) for 48 h. Scale bar: 50 μm. The data are presented as the mean ± SD; *P < 0.05, **P < 0.01, and ***P < 0.001, ****P < 0.0001.

**Fig. 5:**
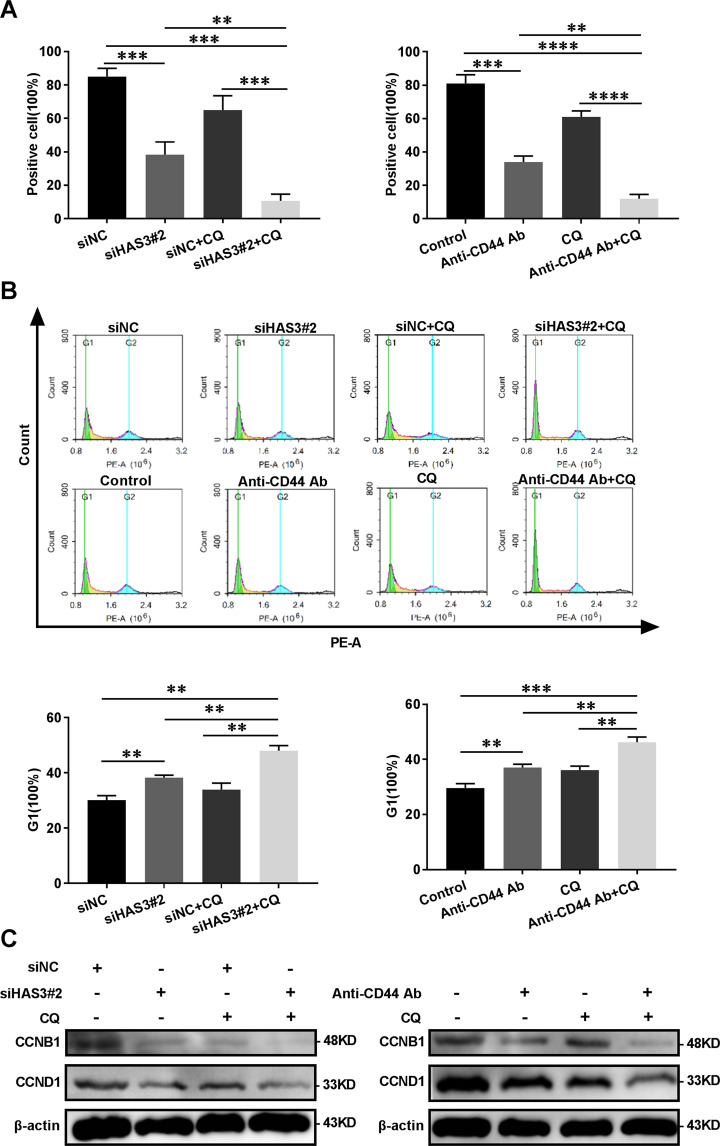
Treatments interfering with HAS3 and CD44 combined with autophagy inhibitors have a synergistic effect on the glioma cell cycle. **A** The percentage of Ki67-positive U251 glioma cells was detected using immunofluorescence staining after transfection with the HAS3 siRNA or culture with a CD44 antibody, followed by treatment with CQ (30 μmol/L) for 48 h. **B** The cell cycle distribution of U251 glioma cells was detected using flow cytometry after transfection with the HAS3 siRNA or culture with a CD44 antibody, followed by treatment with CQ (30 μmol/L) for 48 h. (green: G0-G1, yellow: S, and blue: G2-M). **C** Relative levels of the CCNB1 and CCND1 proteins in U251 glioma cells transfected with the HAS3 siRNA or cultured with a CD44 antibody, followed by treatment with CQ (30 μmol/L) for 48 h. The data are presented as the mean ± SD; *P < 0.05, **P < 0.01, and ***P < 0.001, ****P < 0.0001.

CQ, an inhibitor of autophagy flux, was used to measure autophagy flux in glioma cells^22^ and our research further confirmed that interfering with HA metabolism inhibits cell proliferation by inhibiting autophagy flux. As shown in Fig. 4A, the Western blot results revealed a further increase in levels of the MAP1LC3B-II and P62 proteins following the silencing of HAS3 or treatment with the CD44 antibody in cells treated with CQ. HAS3 silencing or treatment with the CD44 antibody combined with CQ exerted a synergistic effect on reducing the viability of glioma cells and levels of the Ki67 protein (Figs. 4B–D and 5A). Cell cycle arrest is essential for cell proliferation. Therefore, we investigated the effect of silencing HAS3 or treatment with CD44 antibodies on glioma cell cycle arrest. The results of the flow cytometry analysis showed that HAS3 silencing or the CD44 antibody treatment caused cells to remain in G1 phase compared with cells in the control group, and CQ treatment enhanced this effect (Fig. 5B). More importantly, silencing HAS3 or treatment with the anti-CD44 antibodies decreased the levels of the cell cycle-related proteins cyclin B1 and cyclin D1 in glioma cells, and CQ treatment further enhanced this effect (Fig. 5C). Inhibition of HAS3 or treatment with the CD44 antibody combined with autophagy inhibitors exerted synergistic inhibitory effects on glioma proliferation through a molecular mechanism that involves arresting the cell cycle in G1 phase.

### 4-MU suppresses glioma proliferation by blocking autophagy flux, further affecting the cell cycle in vitro and in vivo

**Fig. 6:**
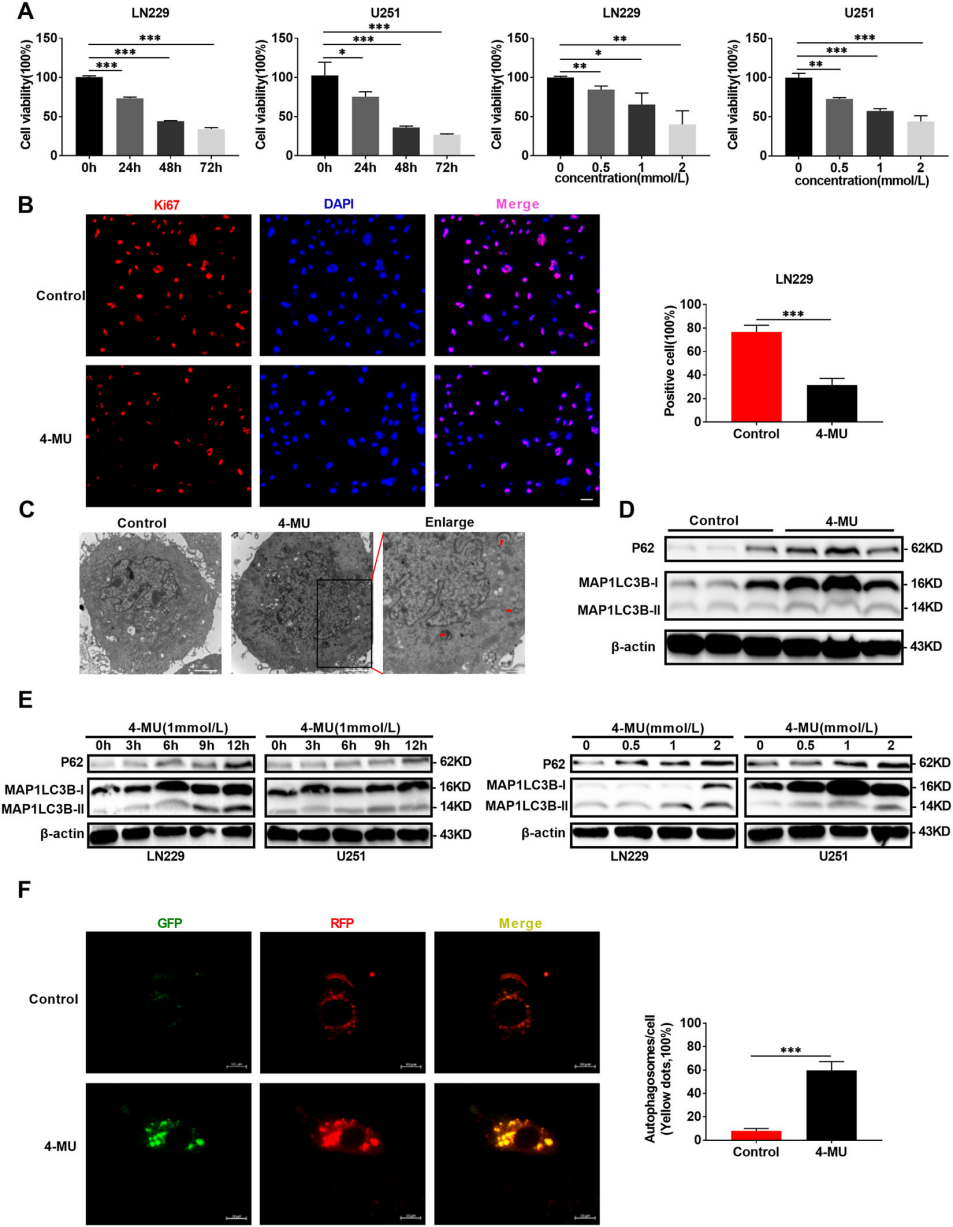
4-MU decreases glioma viability and blocks autophagy flux. **A** Viability of U251 and LN229 glioma cells treated with 4-MU (1 mmol/L) for 0, 24, 48, and 72 h. Viability of U251 and LN229 glioma cells treated with different concentrations of 4-MU (0, 0.5, 1, or 2 mmol/L) for 48 h. **B** Levels of the Ki67 protein in LN229 glioma cells were detected using immunofluorescence staining after treatment with 4-MU for 48 h. Scale bar: 50 μm. **C** TEM images of U251 glioma cells treated with 4-MU for 48 h. The scale bars represent 2 µm in the original image and 0.8 μm in the enlarged image. **D** Relative levels of the P62 and MAP1LC3B proteins in the orthotopic xenograft tumors from the control and 4-MU-treatment groups. **E** Relative levels of the P62 and MAP1LC3B proteins in U251 and LN229 glioma cell lines treated with 4-MU (1 mmol/L) for 0, 3, 6, 9, and 12 h. Relative levels of the P62 and MAP1LC3B proteins in U251 and LN229 glioma cell lines treated with different concentrations of 4-MU (0, 0.5, 1, or 2 mmol/L) for 48 h. **F** GFP-RFP-LC3 fluorescence assays of U251 glioma cells treated with 4-MU for 48 h. Scale bar: 10 μm. The data are presented as the mean ± SD; *P < 0.05, **P < 0.01, and ***P < 0.001, ****P < 0.0001.

**Fig. 7:**
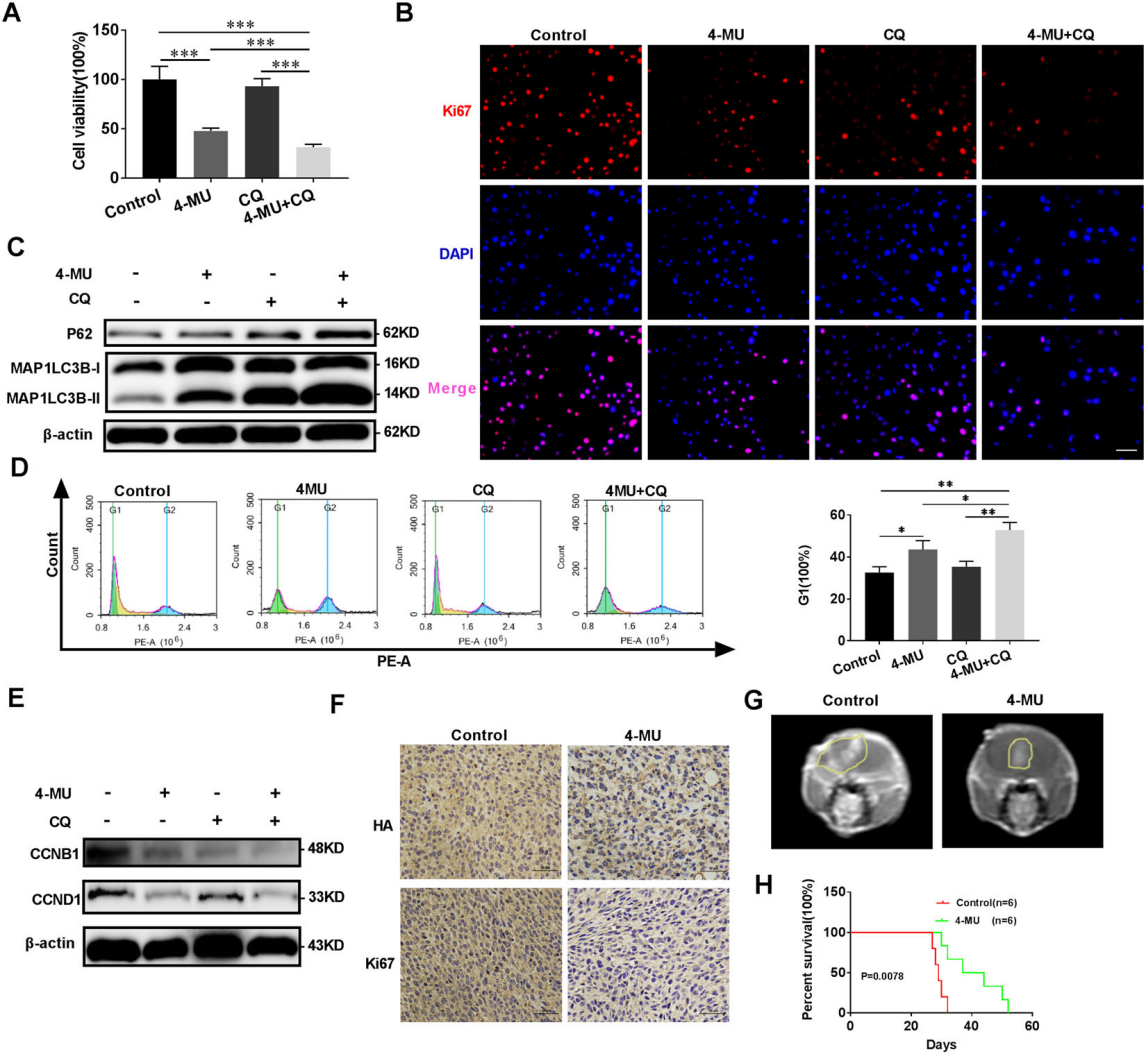
4-MU inhibits glioma growth in vivo and, when combined with autophagy inhibitors, exerts synergistic effects on glioma cell viability, autophagy levels, and the cell cycle. **A** Viability of U251 glioma cells cultured with 4-MU, followed by treatment with CQ (30 μmol/L) for 48 h. **B** Levels of the Ki67 protein in U251 glioma cells were detected using immunofluorescence staining after culture with 4-MU, followed by treatment with CQ (30 μmol/L) for 48 h. **C** Relative levels of the P62 and MAP1LC3B proteins in U251 glioma cells cultured with 4-MU, followed by treatment with CQ (30 μmol/L) for 48 h. Scale bar: 50 μm. **D** The cell cycle distribution was detected in U251 glioma cells using flow cytometry after culture with 4-MU, followed by treatment with CQ (30 μmol/L) for 48 h (green: G0-G1, yellow: S, and blue: G2-M). **E** Relative levels of the CCNB1 and CCND1 proteins in U251 glioma cells cultured with 4-MU, followed by treatment with CQ (30 μmol/L) for 48 h. **F** Representative images of IHC staining for Ki67 and HA in the orthotopic xenograft tumors from the control and 4-MU treatment groups. Scale bar: 50 μm. **G**–**H** Representative MRIs of orthotopic xenograft tumors and survival curves of the control and 4-MU treatment groups. *P*: *P*-value for the comparison of the control and 4-MU groups. The data are presented as the mean ± SD; *P < 0.05, **P < 0.01, and ***P < 0.001, ****P < 0.0001.

UDP is a precursor required for HA synthesis, and 4-MU is a competitive inhibitor of UDP with the ability to penetrate the BBB. In addition, 4-MU inhibited HA synthesis in glioma cells (Supplementary Fig. 3B). Therefore, we examined the effect of 4-MU dissolved in DMSO on glioma cells in vitro and in vivo. As shown in Figs. 6A, 4-MU decreased the viability of U251 and LN229 glioma cells in a dose- and time-dependent manner. Immunofluorescence staining revealed a significant decrease in Ki67 expression in both U251 and LN229 cell lines (Fig. 6B and Supplementary Fig. 3C). Moreover, 4-MU increased the number of autophagic vesicles in vitro (Fig. 6C) and the levels of the MAP1LC3B-II and P62 proteins in vitro and in vivo (Fig. 6D–E). GFP-RFP-LC3 fluorescence assays showed that 4-MU blocked the autophagy flux of glioma cells (Fig. 6F). Exogenous HA reversed these effects of 4-MU on glioma cells (Supplementary Fig. 3D–E). Interestingly, 4-MU combined with CQ also resulted in a synergistic effect on decreasing cell viability and the levels of Ki67, a cell proliferation marker, increasing the levels of the MAP1LC3B-II and P62 proteins, which are autophagy markers, increasing the percentage of cells in the G1 phase, and decreasing the levels of cell cycle-related proteins, such as cyclin B1 and cyclin D1 (Fig. 7A–E and Supplementary Fig. 3F). A subcutaneous tumor model and an orthotopic tumor model were established with U251 glioma cells to further verify the effect of 4-MU on glioma cells. As shown in Fig. 7F–H and Supplementary Fig. 3G–H, 4-MU decreased the volume of glioma tissues, and the levels of HA and Ki67 were also decreased. Importantly, the mice in the 4-MU treatment group lived longer than the mice in the control group. Overall, 4-MU suppressed the proliferation of glioma cells in vitro and in vivo by blocking autophagy flux.

## Discussion

Although many studies have proposed that the interaction between genes and the external environment leads to the occurrence of glioma, the pathogenesis of glioma remains unclear. The metabolic remodeling of tumors and the microenvironment are closely related to tumor progression^23,24^. Previous studies have confirmed that the abnormal accumulation of HA is related to the dysregulated expression of HA synthases and HA degradation enzymes in patients with many pathological conditions, such as cancer, injury, and inflammation^11^. In the present study, we focused on HA synthesis and CD44, a specific HA receptor, rather than HA degradation. It is known that HA synthesis is associated with HA synthases, including HAS1, HAS2, and HAS3. Researchers have observed a specific HAS expression pattern in different tumors. For example, although the expression of both HAS3 and HAS2 is high in metastatic prostate and colon cancer, HAS1 expression is lower in these tumors^8^. Non-aggressive breast cancer cells express high levels of HAS3 and lower levels of HAS2 compared with aggressive breast cancer cells^25,26^. In our study, by analyzing data from the TCGA database, we observed that HAS1 expression was higher in LGG than in GBM, and HAS1 expression did not significantly correlate with the DFS time of patients with LGG. The expression levels of both HAS3 and HAS2 were upregulated in patients with GBM compared with patients with LGG; however, only HAS3 expression negatively correlated with the survival time of patients with LGG and GBM. Moreover, glioma cell viability was not significantly affected when HAS2 was knocked down or HAS1 was overexpressed. The above findings suggested that HAS3 may play a more important role in the biological behavior of glioma cells and the metabolism of HA in glioma cells. Therefore, we chose HAS3 rather than HAS1 and HAS2 in this research.

CD44, a transmembrane glycoprotein, participates in many physiological processes in cancer, including angiogenesis, cell proliferation, and apoptosis. Moreover, CD44 is a crucial receptor for HA. Many extracellular ligands, principally HA, can bind to the CD44 ectodomain, thereby activating the downstream signaling cascades and ultimately promoting cell motility and proliferation^27,28^. For example, the interaction of LMW-HA with CD44 and TLR enhanced the production of IL-1β/IL-8 via the subsequent activation of MyD88/NF-κB and finally promoted the invasiveness of breast cancer cells. HA can promote tumor-associated macrophages to produce growth factors and ECM components through a CD44-dependent pathway^9^. Due to the alternative splicing of the CD44 ectodomain, CD44 has eleven variants that have been described (CD44s, CD44v1–v10), and the expression of the CD44 variants differs in diverse tissues^29^. Therefore, CD44 has a great significance for HA metabolism and tumor progression, which also provides a theoretical basis for CD44 as a potential therapeutic target in tumor treatment. In our study, both high and low concentrations of a CD44 antibody decreased glioma cell viability, while HA only partially reversed this effect caused by a low concentration of the CD44 antibody (3 µg/ml). This finding can be explained by the fact that the combination of HA with CD44 was fully prevented by high concentrations of the CD44 antibody. These results indicated that glioma progression was promoted by CD44 via HA-dependent pathway. However, the previous report that CD44 receptor can also bind to other ligands, thereby promoting cell biological changes. Our results only indicate that HA can play a role through CD44, but it does not deny the biological role of CD44 on glioma cells through HA-independent pathways, which is also a goal of our future research.

Autophagy is a mechanism by which intracellular proteins and organelles are self-degraded by cellular lysosomes. Tumor cells obtain amino acids and other macromolecular substances for biosynthesis through autophagy^30^. Autophagy is a double-edged sword that not only promotes the survival of tumor cells but also induces the death of tumor cells^31^. On the one hand, under stress such as hypoxia or starvation, autophagy can provide tumor cells with metabolic substrates and prolong tumor cell survival. On the other hand, autophagy can inhibit tumor growth through eliminating damaged proteins and organelles, which ultimately prevents genome damage^32^. In addition, researchers have demonstrated that either the inhibition of autophagy or its excessive activation can inhibit glioma cell proliferation^22,33^. As shown in this present study, interference with HA metabolism by inhibiting HAS3, CD44 or the precursor of HA synthesis can block autophagy flux and further arrest the tumor cell cycle in G1 phase, due to blocking autophagy flux leads to the inhibition of late-stage autophagy, we suspected that the inhibition of glioma cell proliferation by blocking HA metabolism is achieved through the inhibition of autophagy rather than its over-activation. Moreover, CQ enhanced the inhibition of glioma cell growth induced by interfering with the HA metabolism pathway. CQ and the inhibition of HA metabolism exert a synergistic inhibitory effect on glioma cell growth.

4-MU inhibits HA synthesis through the depletion of cellular UDP, which finally leads to changes in the tumor microenvironment and the inhibition of tumor growth^34^. 4-MU is a derivative of coumarin, it has been used for choleretic and antispasmodic treatment, and its oral safety has been proven^35^. Recently, research has demonstrated that dietary supplementation of 4-MU can serve as an effective chemotherapeutic for prostate cancer. 4-MU can also decrease the growth of pancreatic cancer by inhibiting HA synthesis^14,36^. The BBB is known to be a major obstacle for glioma treatment; Fortunately, 4-MU has the characteristic of relatively small molecular weight and ability to penetrate the BBB^15^. In our study, treatments interfering with the synthesis of HA by 4-MU inhibited the growth of glioma cells. Taken together, all of these findings suggest that 4-MU could act as a potential chemotherapeutic for glioma treatment in the future.

In summary, treatments interfering with HA metabolism inhibit the malignant behavior of glioma cells by blocking autophagy flux. 4-MU functions as a small HA precursor competitive inhibitor that exerts an anti-glioma effect. Importantly, 4-MU has been clinically proven to be orally safty. Finally, we propose that treatments interfering with HA metabolism and 4-MU may represent effective strategies for glioma treatment in the future.

## Materials and methods

### Patient samples collection

From January 2019 to January 2020, non-tumor brain tissues (*N* = 6), low-grade glioma (LGG; *N* = 10), and glioblastoma multiforme (GBM; *N* = 9) were collected from surgical resection at the Department of Neurosurgery, First Affiliated Hospital of Harbin Medical University. This research was approved by the Ethics Committee of First Affiliated hospital of Harbin Medical University and performed in accordance with the principles of the Declaration of Helsinki. All participants provided written informed consent.

### Cell culture

Human glioma cell lines were obtained from the China Infrastructure of Cell Line Resource (National Science & Technology Infrastructure, NSTI). The glioma cell lines were cultured with Dulbecco’s Modified Eagle’s Medium (DMEM; 10013CVRC, Corning, USA) containing 10% fetal bovine serum (FBS; 16000-044, Gibco, USA) in an incubator at 37 °C with a 5% CO_2_ atmosphere.

### MTT assay

Glioma cells were cultured in 96-well dishes (JET BIOFIL, China) at a density of 4000 cells/well. After treating the cells under different conditions, 10 μl of MTT (5 mg/ml) was added to each well, and the cells were cultured for 4 h at 37 °C in the presence of 5% CO_2_. Next, the culture medium was replaced with 150 μl of dimethyl sulfoxide. Cell viability was measured by recording the absorbance at a wavelength of 490 nm using a BioTek ELx800 (USA) microplate reader according to the manufacturer’s instructions. MTT (Cat# HY-15924), chloroquine (CQ; Cat# HY-17589A) and hyaluronic acid sodium (Cat# HY-B0633) were purchased from MedChemExpress, and 4-MU (Cat# M1381) was purchased from Sigma-Aldrich. The CD44 antibody (Cat# 217594-100ULCN) was purchased from Millipore.

### Cell transfection

The Lipofectamine 2000 reagent (Cat# 11668019, Invitrogen, USA) was used to transfect the glioma cell lines with siRNAs/plasmid according to the manufacturer’s instructions. The siRNAs and over-expression plasmids were purchased from GENERAL BIOSYSTEMS (China). Lentiviruses (shNC, shHAS3, and shCD44) were purchased from Wanleibio (China).

### Quantitative real-time PCR (qRT-PCR)

Total RNA was extracted using TRIzol reagent (Cat# 15596026, Invitrogen, USA) and reverse transcribed using a Roche Transcriptor cDNA Synthesis Kit (Cat #4897030001) according to the manufacturer’s specifications. A SYBR Green PCR Master Mix kit (Cat# 4913914001, Roche, Germany) was utilized to verify the expression of the target gene. qRT-PCR was performed using an ABI Prism 7500 fast thermocycler (Applied Biosystems, CA, USA). The sequences of the GAPDH, HAS1, HAS2, HAS3, and CD44 primers are shown in Table 1.

**Table. 1:** Sequence of siRNA, prismer and lentivirus.

*siRNA sequence*
siNC 5′GUA UGA CAA CAG CCU CAA GTT3′
HAS2-siRNA1 5′GGG CAC AUC AGG AAG GAA ATT3′
HAS2-siRNA2 5′AGU CAU GGG CAG AGA CAA ATT3′
HAS2-siRNA3 5′CUA UGU AUC CUG AGA AUA ATT3′
HAS3-siRNA1 5′CAU CAG AAG UUC CUA GGC ATT3′
HAS3-siRNA2 5′GGC UAC CGA ACU AAG UAU ATT3′
HAS3-siRNA3 5′CUA UAC UGU AUG GCU GCU ATT3′
CD44-siRNA1 5′ GGA CCA AUU ACC AUA ACU ATT3′
CD44-siRNA2 5′CUC CCA GUA UGA CAC AUA UTT3′
CD44-siRNA3 5′GCA GUC AAC AGU CGA AGA ATT3′
*Prismer sequence*
GAPDH F-5′ CACCCACTCCTCCACCTTTGA3′, R-5′ACCACCCTGTTGCTGTAGCCA3′
HAS1 F-5′ GAGCCTCTTCGCGTACCTG3′, R-5′ CCTCCTGGTAGGCGGAGAT3′
HAS2 F-5′ CTCTTTTGGACTGTATGGTGCC3′, R-5′ AGGGTAGGTTAGCCTTTTCACA3′
HAS3 F-5′TTACTTCCGGGAGTGGCTCTACAAC3′, R-5′CGTCAGCAGGAAGAGGAGAATG3′
CD44 F-5′TGGAAGATTTGGACAGGACAG3′,R-5′CGTGTGTGGGTAATGAGAGGTA3′
*Lentivirus sequences*
LV-shNC 5′GUA UGA CAA CAG CCU CAA GTT3′
LV-shHAS3 5′GGC UAC CGA ACU AAG UAU ATT3′
LV-shCD44 5′GGA CCA AUU ACC AUA ACU ATT 3′

### Western blot analysis

Protein samples were extracted from glioma cell lines/glioma tissues. Protein samples were separated on 12.5% SDS-PAGE gels and transferred to PVDF membranes. The membranes were blocked with 5% skim milk and incubated with the primary antibody at 4 °C overnight. The next day, the membranes were incubated with fluorescent dye-conjugated secondary antibodies at room temperature for 2 h. The membranes were observed using a ChemiDoc XRS + Imaging System (Bio-Rad, USA). The following primary antibodies were utilized in the present study: P62 (18420-1-AP, Proteintech); MAP1LC3B (L7543, Sigma); CCND1 (AF0931, Affinity); CCNB1 (DF6786, Affinity); HAS3 (DF13055, Affinity); CD44 (A0340, Abclonal); and β-actin (TA-09, ZSGB-BIO).

### ELISA

An ELISA kit (Cat# 10800) was purchased from Shanghai Jianglai Industrial Limited By Share Ltd. Glioma cells or tissues were treated with RIPA lysis buffer on ice. After centrifugation, the supernatants from glioma cells or tissues were obtained. Fifty microliters of supernatant and standard samples were added to each well. Horseradish peroxidase (HRP) (100 μl) was added to each well, and the cells were incubated at 37 °C for 1 h. The primary liquid was replaced with a scrubbing solution and dried using absorbent paper by patting the plate five times. Substrates A (50 μl) and B (50 μl) were added to each well, and the samples were incubated in the dark for 15 min at 37 °C. Stop buffer (50 μl) was then added to each well. Finally, the optical density (OD) was measured at a wavelength of 450 nm using a BioTek elx800 (USA) microplate reader according to the manufacturer’s instructions.

### Transmission electron microscopy (TEM)

Cells immobilized with 2.5% glutaraldehyde at 4 °C were postfixed with 1% osmium tetroxide, followed by dehydration in increasing concentrations of ethanol and acetone. Autophagic vesicles were observed using TEM.

### GFP-RFP-LC3 fluorescence analysis

Glioma cells were cultured in 6-well dishes (JET BIOFIL, China) at a density of 3–5 × 10^4^ cells/well. When the cell density reached 50–60%, the lentivirus was removed from storage at −80 °C and thawed at 4 °C. Then, the culture medium was removed from the six-well plate, and the cells were washed three times with PBS; Fresh serum-free medium (1 ml) and lentiviruses (20 µl) solution was added to the six-well dishes. A control group was also established. After 24 h of infection, cell growth and fluorescence were observed, and subsequent experiments were performed. GFP-RFP-LC3 lentivirus was purchased from Shanghai Genechem (China).

Autophagy flux was observed according to the manufacturer’s protocol. After treatment under different conditions, fluorescence was observed using a confocal microscope, in which yellow dots represented autophagosomes, and red dots represented autolysosomes.

### Flow cytometry analysis of the cell cycle

Cells were collected and fixed with 70% ethyl alcohol for 24 h. Next, the cells were stained with a cell cycle analysis kit (Beyotime P0010, China) at 4 °C for 30 min. The cell cycle results were detected using a flow cytometer.

### Immunohistochemical (IHC) staining

The tissues were embedded in paraffin, sliced, and incubated with primary and secondary antibodies to visualize the target protein under the microscope. The main antibodies utilized in the present study were as follows: HAS3 (BF0681, Affinity), CD44 (A0340, Abclonal), HA (Ab53842, Abcam), and Ki67 (A2094-100, Abclonal).

### Mouse tumor model

BALB/C nude mice were purchased from Beijing Vital River Laboratory Animal Technology Co., Ltd. (China). For the subcutaneous tumor model, the mice were divided into control and experimental groups. Serum-free DMEM (100 μl) containing 5 × 10^6^ cells was injected subcutaneously at 1 cm. Tumor volume was measured twice weekly, and the mice were sacrificed 23 days later. The tumor volume was calculated using the formula (width)2 × (length)/2. For orthotopic transplantation, the mice were divided into control and experimental groups. Serum-free DMEM (5 µl) containing 2 × 10^6^ cells was injected into the mouse brain. The injection location was 2.5 mm to the right of the midline and 0.5 mm behind the coronal suture at a depth of 3.5 mm. After 21 days, the two groups were scanned using MRI. At 52 days, the nude mice with residual orthotopic tumors were euthanized. Finally, the survival curve was generated according to the time of death of the mice. After the successful establishment of the mouse tumor model, the mice were randomly divided into the control group and treatment group, and the mouse breeder was blinded to the control and treatment groups during the experiment. All animal studies were approved by the Ethics Committee of First Affiliated Hospital of Harbin Medical University and performed in accordance with the principles of the Declaration of Helsinki.

### Bioinformatics analysis

The Cancer Genome Atlas (TCGA) database was used to draw a curve showing the expression of target genes in patients with LGG and GBM. According to the TCGA database, we generated disease-free survival (DFS) curves for target genes in patients with LGG and GBM. Gene expression data for 516 patients with LGG and 154 patients with GBM and DFS data for 476 patients with LGG and 94 patients with GBM were included. The top 75% was chosen for the demarcation point. The data from TCGA were downloaded from https://xenabrowser.net/datapages/?hub=https://tcga.xenahubs.net:443.

### Statistical analysis

Statistical analyses comparing data between groups were performed using Student’s *t*-test or one-way ANOVA (Prism software version 7.0). *P* < 0.05, <0.01, <0.001, <0.0001 were denoted in graphs by different asterisks.

## Supplementary information

Supplementary figure legends

Supplementary figure 1

Supplementary figure 2

Supplementary figure 3
