# Supplementary material for: Interfering with hyaluronic acid metabolism suppresses glioma cell proliferation by regulating autophagy
Source: Cell Death Dis. 2021 May 13;12(5):486. doi: 10.1038/s41419-021-03747-z (PMC8119697; doi:10.1038/s41419-021-03747-z)
Supplement: Supplementary file 1 — Supplementary figure legends [file 41419_2021_3747_MOESM1_ESM.docx]

**Supplementary figure legends**

**Supplementary Fig. 1. The expression and survival curves of HAS1 and HAS2 different grades of glioma. (A-B)** Relative expression of the HAS1 and HAS2 mRNAs in patients with LGG and GBM from TCGA. Survival curves of HAS1 and HAS2 for patients with LGG and GBM from TCGA database. **(C)** Viability of U251 and LN229 glioma cells transfected with HAS1 over expression plasmid or HAS2 siRNA for 48 h. **(D)** Relative concentration of HA in five glioma cell lines (LN229, U251, U87, T98, A172) and HUVEC cells by ELISA. **(E)** Relative expression of the HAS3 mRNA in five glioma cell lines (LN229, U251, U87, T98, A172) and HUVEC cells. **(F)** Relative expression of the CD44 mRNA in five glioma cell lines (LN229, U251, U87, T98, A172) and HUVEC cell. **(G)** Relative levels of the HAS3 and CD44 proteins in five glioma cell lines (LN229, U251, U87, T98, A172) and HUVEC cells. The data are presented as the mean ± SD; *P < 0.05, **P < 0.01, and ***P < 0.001, ****P< 0.0001.

**Supplementary Fig. 2. Interfering with HAS3 and CD44 suppresses glioma cell proliferation in vitro and in vivo. (A)** Relative concentration of HA in U251 and LN229 glioma cells transfected with the HAS3 siRNA by ELISA. **(B)** Viability of U251 glioma cells cultured with the CD44 antibody (3 µg/ml), followed by treatment with HA (25 µg/ml) for 48 h. **(C)** Levels of the Ki67 protein in U251 glioma cells were detected using immunofluorescence staining after transfection with HAS3 siRNA or treatment with a CD44 antibody for 48 h. Scale bar: 50 μm. **(D)** Viability of U251 and LN229 glioma cells transfected with CD44 over expression plasmid for 48 h. Viability of U251 and LN229 glioma cells transfected with HAS3 over expression plasmid for 48 h. **(E)** Gene knockout efficiency of the HAS3 siRNA and CD44 siRNA. **(F)** Representative images of the subcutaneous xenograft tumors from the control and lentivirus HAS3 siRNA or lentivirus CD44 siRNA stably transfected glioma cell groups. **(G)** The tumor volume of the subcutaneous xenograft tumors from the control and lentivirus HAS3 siRNA or lentivirus CD44 siRNA stably transfected glioma cell groups. The data are presented as the mean ± SD; *P < 0.05, **P < 0.01, and ***P < 0.001, ****P< 0.0001.

**Supplementary Fig. 3. 4-MU suppresses glioma cell proliferation in vitro and in vivo.** **(A)** Relative levels of the P62 and MAP1LC3B proteins in U251 glioma cells transfected with the HAS3 siRNA, followed by treatment with HA (25 µg/ml) for 48 h. **(B)** Relative concentration of HA in U251 and LN229 glioma cells treated with 4-MU for 48 h by ELISA. **(C)** Levels of the Ki67 protein in U251 glioma cells were detected using immunofluorescence staining after treatment with 4-MU for 48 h. Scale bar: 50 μm. **(D)** Viability of U251 glioma cells cultured with 4-MU, followed by treatment with HA (25 µg/ml) for 48 h. **(E)** Relative levels of the P62 and MAP1LC3B proteins in U251 glioma cells cultured with 4-MU, followed by treatment with HA (25 μg/ml) for 48 h. **(F)** The percentage of Ki67-positive U251 glioma cells cultured with 4-MU, followed by treatment with CQ (30 μmol/L) for 48 h. **(G-H)** Representative images and volumes of the subcutaneous xenograft tumors from the control and 4-MU treatment groups. The data are presented as the mean ± SD; *P < 0.05, **P < 0.01, and ***P < 0.001, ****P< 0.0001.
