# Supplementary figures and images for: Interfering with hyaluronic acid metabolism suppresses glioma cell proliferation by regulating autophagy
Source: Cell Death Dis. 2021 May 13;12(5):486. doi: 10.1038/s41419-021-03747-z (PMC8119697; doi:10.1038/s41419-021-03747-z)

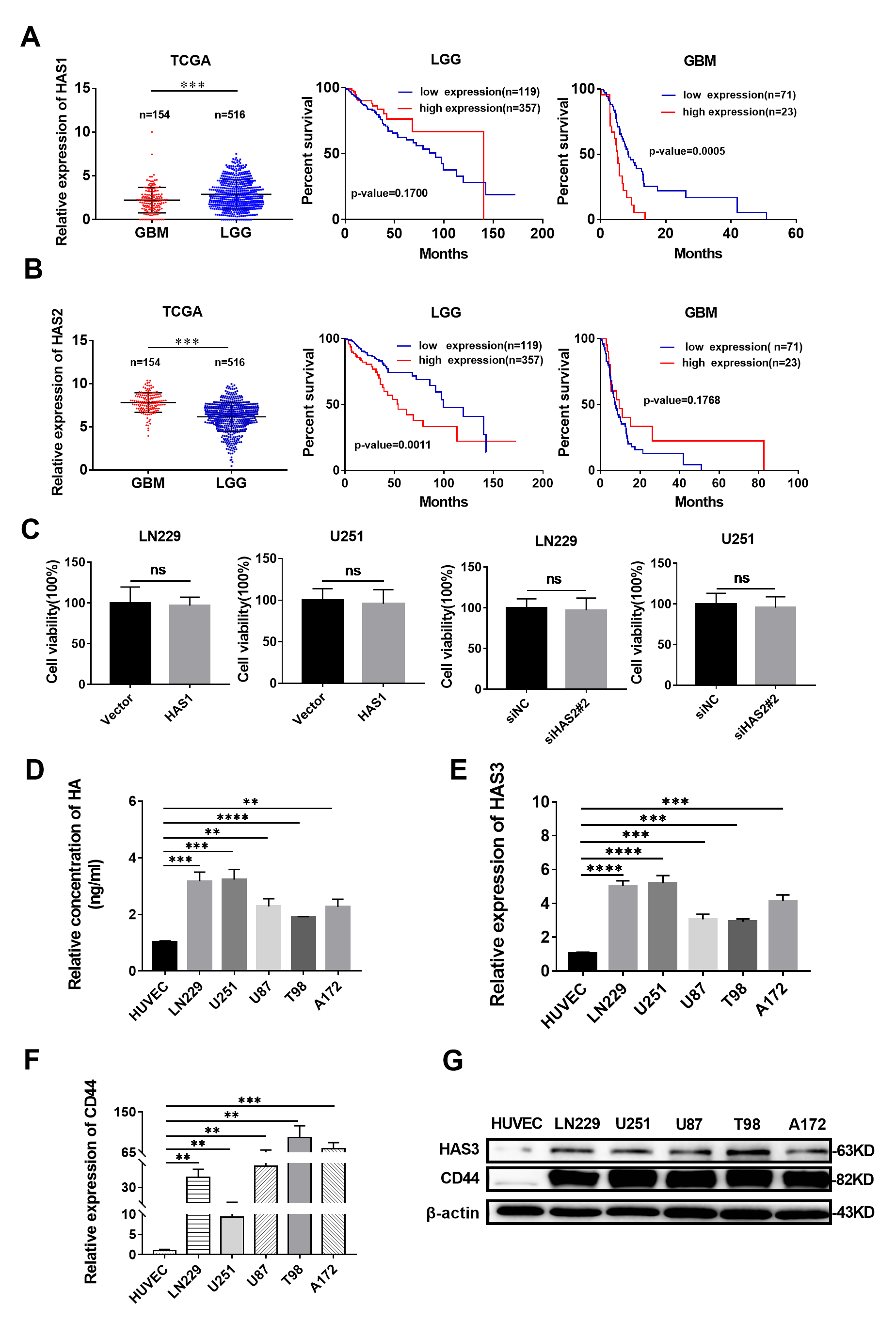

Supplement: Supplementary file 2 — Supplementary figure 1 [file 41419_2021_3747_MOESM2_ESM.tif]

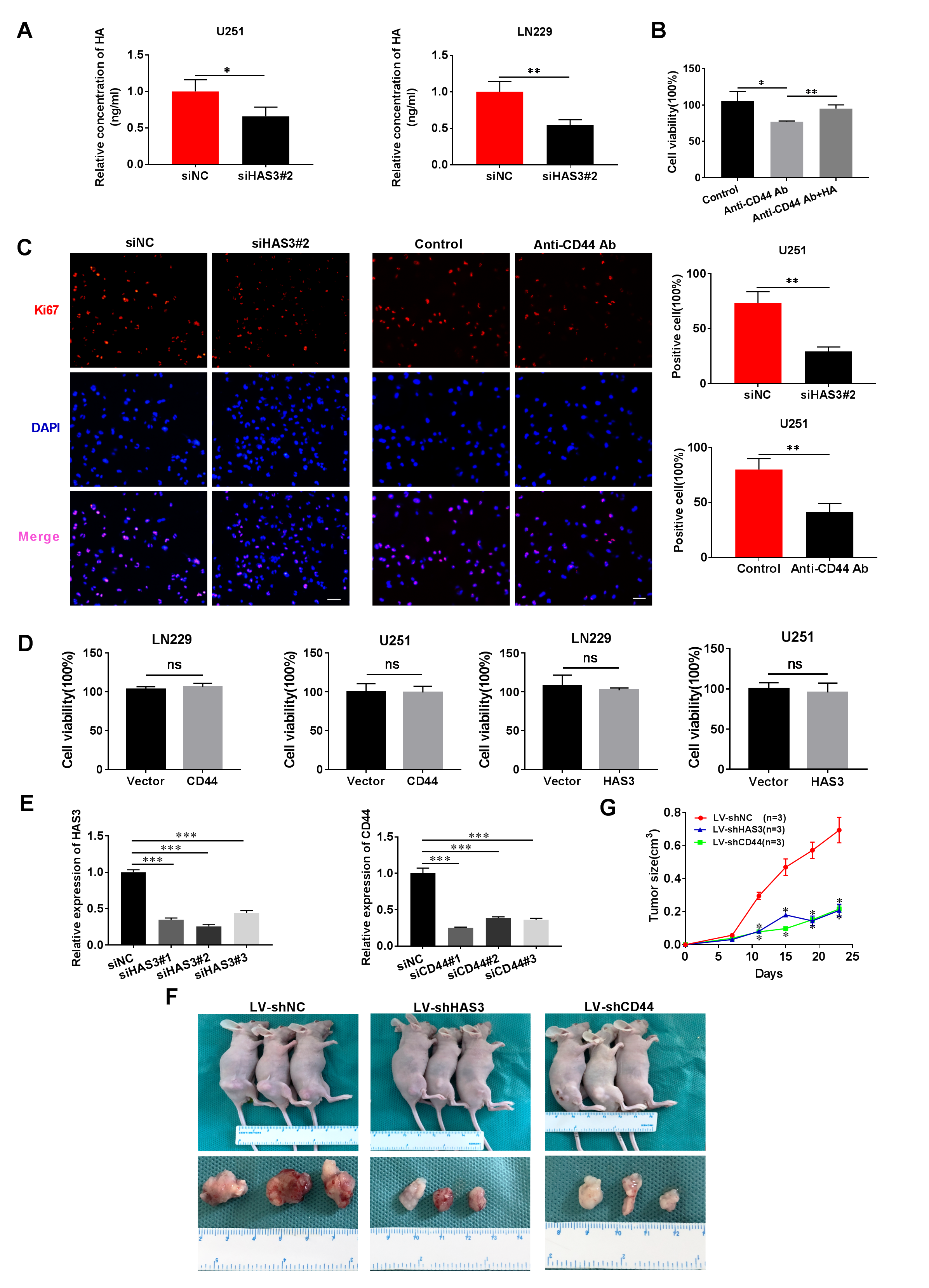

Supplement: Supplementary file 3 — Supplementary figure 2 [file 41419_2021_3747_MOESM3_ESM.tif]

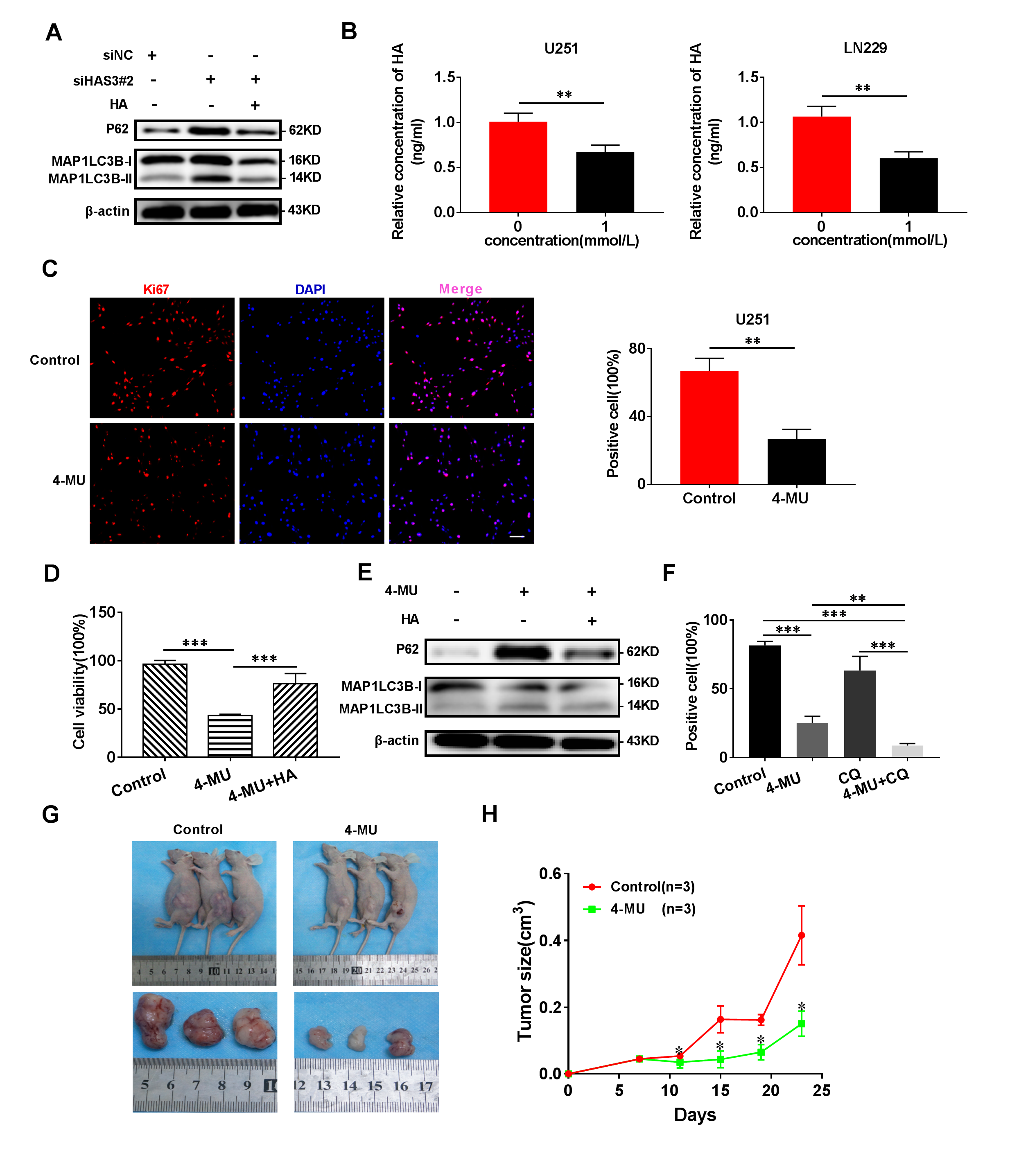

Supplement: Supplementary file 4 — Supplementary figure 3 [file 41419_2021_3747_MOESM4_ESM.tif]
